# Hypothalamic–pituitary–bone marrow axis promotes tumor-induced immunosuppression

**DOI:** 10.1093/lifemedi/lnac058

**Published:** 2022-12-15

**Authors:** Yueli Xu, Jiaxian Yan, Rongbin Zhou

**Affiliations:** Hefei National Research Center for Physical Sciences at the Microscale, the CAS Key Laboratory of Innate Immunity and Chronic Disease, School of Basic Medical Sciences, Division of Life Sciences and Medicine, University of Science and Technology of China, Hefei 230027, China; Hefei National Research Center for Physical Sciences at the Microscale, the CAS Key Laboratory of Innate Immunity and Chronic Disease, School of Basic Medical Sciences, Division of Life Sciences and Medicine, University of Science and Technology of China, Hefei 230027, China; Hefei National Research Center for Physical Sciences at the Microscale, the CAS Key Laboratory of Innate Immunity and Chronic Disease, School of Basic Medical Sciences, Division of Life Sciences and Medicine, University of Science and Technology of China, Hefei 230027, China; Insitute of Health and Medicine, Hefei Comprehensive National Science Center, Hefei 230601, China

Tumors can induce immunosuppression by promoting immunosuppressive cells to accumulate around the tumor immune microenvironment (TIME) [1] and suppress the activation of effector T lymphocytes by blocking immune checkpoints (i.e., PD-1 and CTLA-4), which leads to evasion of tumor immune surveillance and attack [2]. Immune checkpoint blockade (ICB) has emerged as an important strategy for immunotherapy, which could restore the function of immune. Although ICB has great clinical achievements, approximately 70% of patients fail to respond to it. Therefore, it is necessary to further elucidate the cellular and molecular mechanisms of tumor-induced immunosuppression.

Cancer patients often endure mental or emotional stress such as depression, fear, and anxiety, and epidemiological studies have found that chronic depression and stress accelerate tumor development and weaken the effects of tumor immunotherapy [3], suggesting that the nervous system plays an important role in tumor growth and immune regulation. The neuroendocrine system is the main pathway of the central nervous system that regulates the immune response and is mainly composed of the hypothalamus and pituitary gland. The neuroendocrine system has been reported to regulate immune responses by producing hormones, such as adrenocorticotropic hormone, thyroid stimulating hormone (TSH), prolactin, and sex hormones. Moreover, previous studies have reported that some downstream hormones or effectors, such as glucocorticoids, estrogen, androgen, and progesterone, are elevated in cancer patients and can regulate the function of immune cells [3–7], suggesting that the neuroendocrine system might modulate tumor immunity. However, the function of the neuroendocrine system in tumor immunosuppression is unclear.

Here, we found that the pituitary hormone α-melanocyte-stimulating hormone (α-MSH) promotes tumor-induced myelopoiesis and immunosuppression. Subcutaneous implantation of different tumors could lead to hypothalamus activation and the production of α-MSH by pituitary. α-MSH acted on melanocortin 5 receptor (MC5R)-expressing bone marrow progenitors to promote myelopoiesis, myeloid cell accumulation, and immunosuppression, which could promote tumor growth. Meanwhile, inhibiting or antagonizing MC5R boosted antitumor immunity and enhanced ICB therapy [8].

First, we constructed different tumor models, including both ICB-resistant (LLC and B16F10-GMCSF) and ICB-sensitive tumors (MC38 and MCA205), to explore the role of the hypothalamic–pituitary (HP) unit in tumor immunity. We found that the production of α-MSH was increased in the serum of these tumor-bearing mice, but the production of other pituitary hormones was normal, including β-endorphin, TSH, prolactin, follicle-stimulating hormone, and luteinizing hormone. Meanwhile, we found that tumor transplantation resulted in the activation of neurons in the paraventricular nucleus of the hypothalamus and increased POMC expression in the intermediate lobe of the pituitary gland. These results indicate that tumor bearing in mice promotes hypothalamus activation and pituitary α-MSH production.

Next, we investigated the role of POMC and pituitary-derived α-MSH in tumor immunity, and used stereotaxic injection of adenoviral vectors to knock down the expression of the pituitary *Pomc* gene. The results showed that knockdown of pituitary *Pomc* expression significantly inhibited the growth of different tumors. In addition, we found that knockdown of pituitary *Pomc* expression could enhance antitumor immunity, and inhibit myelopoiesis and the accumulation of tumor-associated myeloid cells (MDSCs and TAMs). In conclusion, these results indicate that α-MSH suppresses antitumor immunity by regulating myelopoiesis.

To investigate the mechanism by which α-MSH promotes myelopoiesis and immunosuppression, we examined the expression of α-MSH receptors and found that MC5R is highly expressed on LSK cells in BM. By constructing *Mc5r*-systemic or conditional-deficient mice for the tumor-bearing experiment, we found that *Mc5r* deficiency significantly enhanced antitumor immunity and inhibited the development of different tumor types. α-MSH-MC5R promoted the proliferation of BM-LSK cells via the ERK-STAT3 pathway. In addition, blocking MC5R with antagonists could inhibit tumor growth and exert synergistic effects with immune checkpoint drugs.

Finally, we found that the serum α-MSH concentration is elevated and correlates with circulating MDSCs in cancer patients, such as those with non–small cell lung cancer and head and neck cancer.

**Figure 1.**
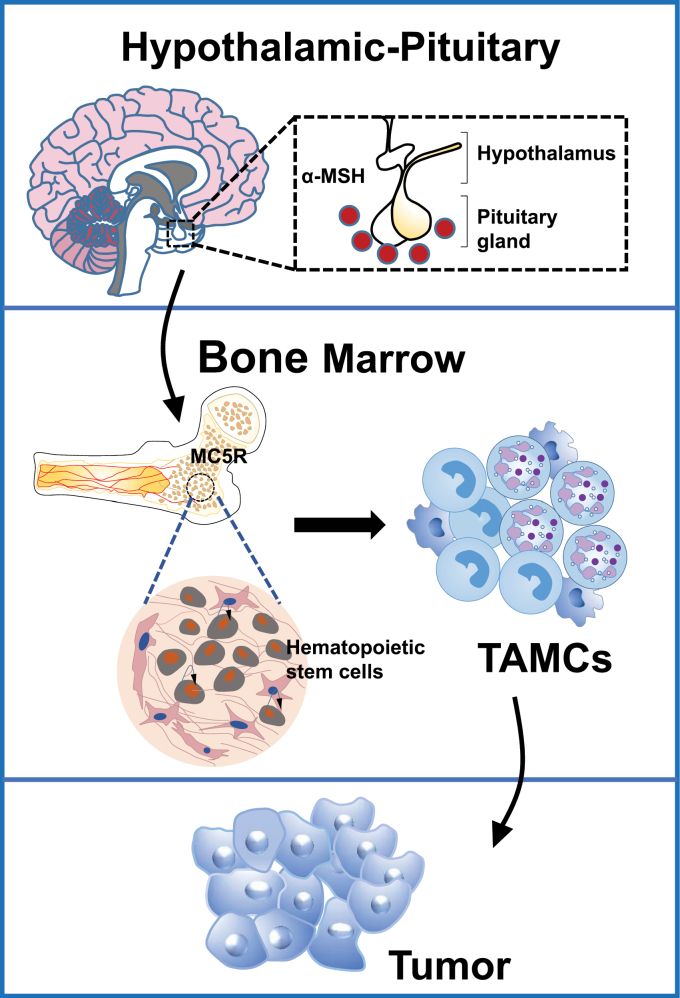
Schematic mechanism of the hypothalamic–pituitary–bone marrow axis promoting tumor-induced myelopoiesis and immunosuppression.

In conclusion, our results indicate that tumors can induce the activation of neurons in the hypothalamus and pituitary hormones, which then suppress tumor immunity and promote tumor growth (Fig. 1). Thus, these results reveal the hypothalamic–pituitary–bone marrow axis as a novel neuroendocrine pathway that contribute to tumor-induced immunosuppression and suggest that MC5R is a potential new target for cancer immunotherapy.

Although our results demonstrate that tumor transplantation can promote hypothalamus activation, the mechanism remains unclear. A possible explanation is that TIME can produce some cytokines (IL-1β, IL-6, TNF-α, etc.) that can cross the blood–brain barrier and then directly or indirectly activate the hypothalamus. Another possible explanation is that TIME-derived inflammatory substances can affect sensory neurons and then convey the signal to hypothalamus.
